# Barriers to influenza vaccination among different populations in Shanghai

**DOI:** 10.1080/21645515.2020.1826250

**Published:** 2020-12-03

**Authors:** Sijin Yan, Yuanping Wang, Weiping Zhu, Li Zhang, Huozheng Gu, Dan Liu, Aiqin Zhu, Hongmei Xu, Lipeng Hao, Chuchu Ye

**Affiliations:** Division of Infectious Disease Prevention and Disinfection Management, Shanghai Pudong New Area Center for Disease Control and Prevention, Shanghai, China

**Keywords:** Influenza, vaccination, coverage, children, adults, older people

## Abstract

Background: Seasonal influenza vaccination coverage remains low in most areas of China. Its influencing factors and barriers in various populations receiving influenza vaccinations need to be well understood to promote vaccination. Methods: A cross-sectional survey was conducted with residents in 48 communities. Vaccination status in the 2018–2019 influenza season and reasons for or against vaccination were surveyed. The potential factors influencing vaccination uptake were determined using bivariate logistic regression. Results: In total, 1301 of the 11053 respondents received an influenza vaccine during the 2018–2019 season with a coverage rate of 11.8% (95% CI, 11.2–12.4). The vaccine coverage was highest among children (26.6%, 95%CI: 24.8–28.5), followed by adults (8.2%, 95%CI: 7.4–9.0) and elderly people (7.3%, 95%CI: 6.5–8.1) (*p* < .001). Those with chronic underlying conditions all had higher vaccine coverage than did those without for different groups (*p* < .001). Among the three groups, the most common reason for being unvaccinated was worrying about the side effects (45.0%), believing they were healthy and did not need to get vaccinated (42.2%), and lack of influenza vaccine awareness (48.3%). Low education level and lack of awareness were identified as predictors of low coverage rate. Conclusion: Influenza vaccination coverage is low among different populations in Shanghai. Our study highlights the need for appropriate influenza vaccination strategies and programmes targeting different populations.

## Introduction

Seasonal influenza is an acute respiratory infection caused by influenza viruses that circulate in all parts of the world. Worldwide, annual influenza epidemics are estimated to result in about 3 to 5 million cases of severe illness, and about 290 000 to 650 000 respiratory deaths.^1^ Seasonal influenza is estimated to cause 88,100 respiratory deaths annually in China.^2^ Although influenza vaccination is the most effective way to prevent influenza infection,^1^ vaccination coverage rates (VCRs) were only 1.5–2% between 2004 and 2014 in a national survey in China.^3^

Shanghai is a major city in eastern China and is one of the most developed cities in the world. In recent years, a few studies have been conducted in Shanghai to understand the current influenza vaccination coverage rate (VCR) as well as to consider efficient policies to increase it among specific groups, including older individuals, nurses and those with chronic obstructive pulmonary disease (COPD).^4–6^ However, comparison of the coverage rate and influencing factors of the influenza vaccine among different populations in Shanghai have not been well explored or discussed. More studies should be conducted to improve the vaccine coverage rate for influenza prevention.

This study aimed to assess the influenza VCR among different populations in Shanghai during the 2018–2019 influenza season and to determine the reasons for vaccination or non-vaccination. The findings of this study are intended to provide scientific evidence for the implementation of appropriate strategies and programmes for different populations.

## Materials and methods

### Study design and sampling procedure

A complex sampling method was used to recruit survey respondents. Each referral area of the 48 community health centers in Pudong New Area randomly selected one community, and all residents living in the selected community were eligible for the interview. Trained community health workers traveled to the selected communities to conduct face-to-face surveys at residential areas or children’s vaccination clinics from Monday to Sunday during day time (from 9 A.M. to 5 P.M.) in our study period. Study subjects were recruited at these locations using a convenience (accidental) sampling method, in which community residents close at hand were interviewed. In order to improve the accuracy of the convenience sampling, the demographic profile of the residents selected was standardized, including a representative gender composition. Because of the different methodologies used, the interviews were conducted separately for three age strata: children <15 years of age, adults between 15 and 60 years of age and elderly people ≥60 years of age. For children, their parents/guardians were responsible for the interviews; adults themselves were investigated; for the elderly, the interviews were conducted by themselves or by their children. The guardians of children or the elderly were not included in our study.

A minimum of 40 completed interviews from children, 65 completed interviews from adults and 75 completed interviews from older people were required for each selected community based on the total sample size of the study. For children <15 years old, a target sample of 1778 was required. This number was calculated based on an assumed influenza vaccination rate of 20% for children aged <15 years.^7^ For adults, a target sample size of 3095 was required based on an assumed influenza vaccination rate of 6%.^7^ A target sample size of 3601 for elderly people ≥60 years of age was required, which was calculated based on an assumed influenza vaccination rate of 5.2%.^4^

### Data collection

A standardized questionnaire was used to collect data from the study subjects. The questionnaires included three sets of questions: (i) socio-demographic characteristics, including age, gender, occupation (of parents or guardians for children), educational attainment and average monthly household income; (ii) self-reported influenza vaccination and reasons for receiving or not receiving the influenza vaccine; and (iii) willingness to vaccinate and related reasons. Written and informed consent was obtained from all participants or from the parents/guardian of children before the interviews.

To validate our results, all interviews were conducted in Mandarin, and questions to child participants were answered by their parents or guardians. A pilot study and training courses for the interviewers were conducted before the study began. An inspector was assigned to each site during the study period to review the ﬁnished questionnaires for completeness and logical errors and to eliminate duplicate surveys. Ineligible questionnaires were returned to the interviewers for veriﬁcation or re-investigation. The collected data were entered into EpiData, version 3.1 (EpiData Association, Odense M, Denmark).

### Statistical analyses

All the analyses were conducted in R, version 3.6.1 or SPSS v21.0. Descriptive statistics analysis was done to show the distribution of demographic characteristics (gender, age, living status, educational attainment and average monthly household income), self-evaluation of health situation and specific underlying diseases. Binary logistic regression models were used to investigate the predictors of influenza vaccination.

## Results

### Characteristics of study population

**Figure 1.**
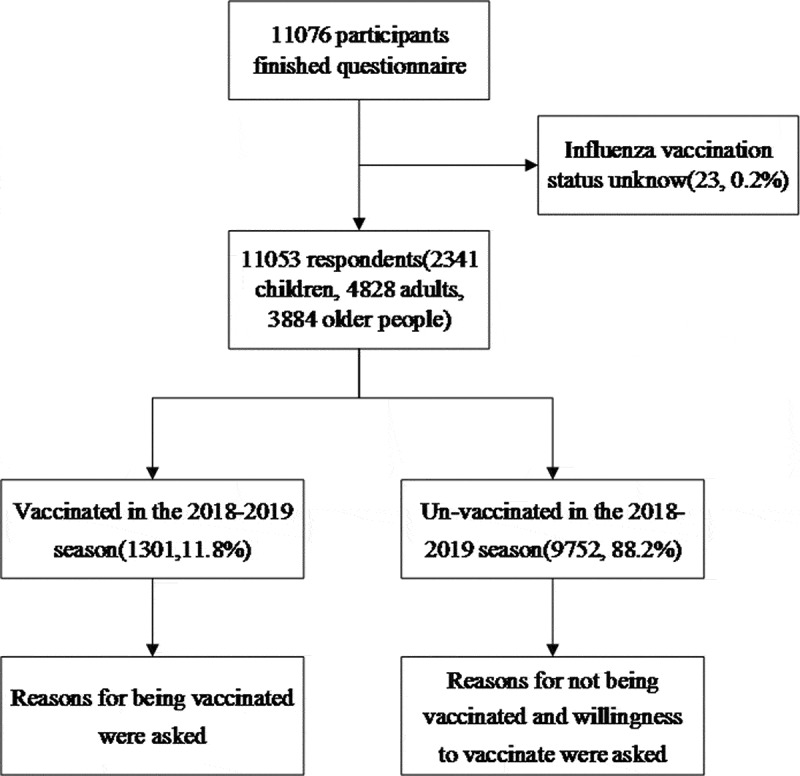
Completion of enrollment survey questions collected in 48 communities in Shanghai, China, 2019

A total of 11076 individuals were interviewed and successfully ﬁnished the questionnaire, including 2346 children, 4839 adults and 3891 older people. Because our study focused on inﬂuenza vaccination, 23 participants whose vaccination status during the 2018–2019 inﬂuenza season was classiﬁed as “unknown” were excluded from the analysis. The final participants included 2341 children, 4828 adults and 3884 older people (Figure 1).

The ages of the respondents ranged from 0.7 to 103.2 years, with a median age of 43.7 [interquartile range (IQR): 18.8–67.1] years, and women accounted for 52.4% of the respondents (Table 1). The majority of the respondents (96.7%) lived with family or friends. More than half of the adults had bachelor’s degrees or above; however, the majority of the elderly (76.3%) had less than a high school-level education. Nearly two-thirds (66.2%) of the participants self-assessed their health status as very healthy/healthy, whereas 25.5%, 7.4% and 5.3% of the individuals suffered from cardiovascular disease, diabetes and chronic respiratory disease, respectively.

**Table 1.** Characteristics of respondents in Pudong New Area, Shanghai, China, 2018–2019

			Different population, n(%)		
		Overall	Children	Adults	Elderly
	Demographic characteristics	N = 11053, n(%)	2341(21.2)	4828(43.7)	3884(35.1)
Gender					
	Male	5260(47.6)	1165(49.8)	2252(46.6)	1843(47.5)
	Female	5793(52.4)	1176(50.2)	2576(53.4)	2041(52.5)
	Age, median (IQR^a^, years)	43.7(18.8–67.1)	7.2(3.7–10.2)	37.5(27.0–48.7)	73.7(66.0–79.8)
	Residence address				
	Shanghai	9388(84.9)	1794(76.6)	3939(81.6)	3655(94.1)
	Other province in China	1665(15.1)	547(23.4)	889(18.4)	229(5.9)
Living status					
	Live alone	366(3.3)	2(0.1)	90(1.9)	274(7.1)
	Live with family or friends	10687(96.7)	2339(99.9)	4738(98.1)	3610(92.9)
Education attainment^b^					
	Primary school	1770(22.1)	–	135(3.3)	1635(42.1)
	Secondary school^c^	2151(26.9)	–	824(20.0)	1327(34.2)
	High school	1373(17.2)	–	702(17.1)	671(17.2)
	Bachelor’s degree or above	2701(33.8)	–	2450(59.6)	251(6.5)
	Self-evaluation of health situation				
	Very healthy	1800(16.3)	693(29.6)	932(19.3)	175(4.5)
	Healthy	5521(49.9)	1383(59.1)	2649(54.9)	1489(38.3)
	Normal	3382(30.6)	261(11.1)	1208(25.0)	1913(49.3)
	Unhealthy	350(3.2)	4(0.2)	39(0.8)	307(7.9)
Monthly household income, (Chinese yuan)^d^					
	<2000	309(2.8)	10(0.4)	36(0.7)	263(6.8)
	2000–4999	1994(18.0)	160(6.8)	594(12.3)	1240(31.9)
	5000–7999	1208(10.9)	247(10.6)	656(13.6)	305(7.9)
	≥8000	1192(10.8)	391(16.7)	648(13.4)	153(3.9)
	Unknown	6350(57.5)	1533(65.5)	2894(60.0)	1923(49.5)
Specific underlying diseases					
	Chronic respiratory diseases	589(5.3)	128(5.5)	135(2.8)	326(8.4)
	Diabetes	816(7.4)	2(0.1)	112(2.3)	702(18.1)
	Cardiovascular disease	2819(25.5)	6(0.3)	505(10.5)	2308(59.4)

^a^IQR: interquartile range.

^b^Children and students were not included.

^c^In China, secondary school education refers to the three-year period between primary school and high school. The ages of secondary school students are approximately 12 to 15 years old.

^d^1 US dollar6.43 Chinese yuan.

### Influenza vaccination coverage rates and their influencing factors

**Figure 2.**
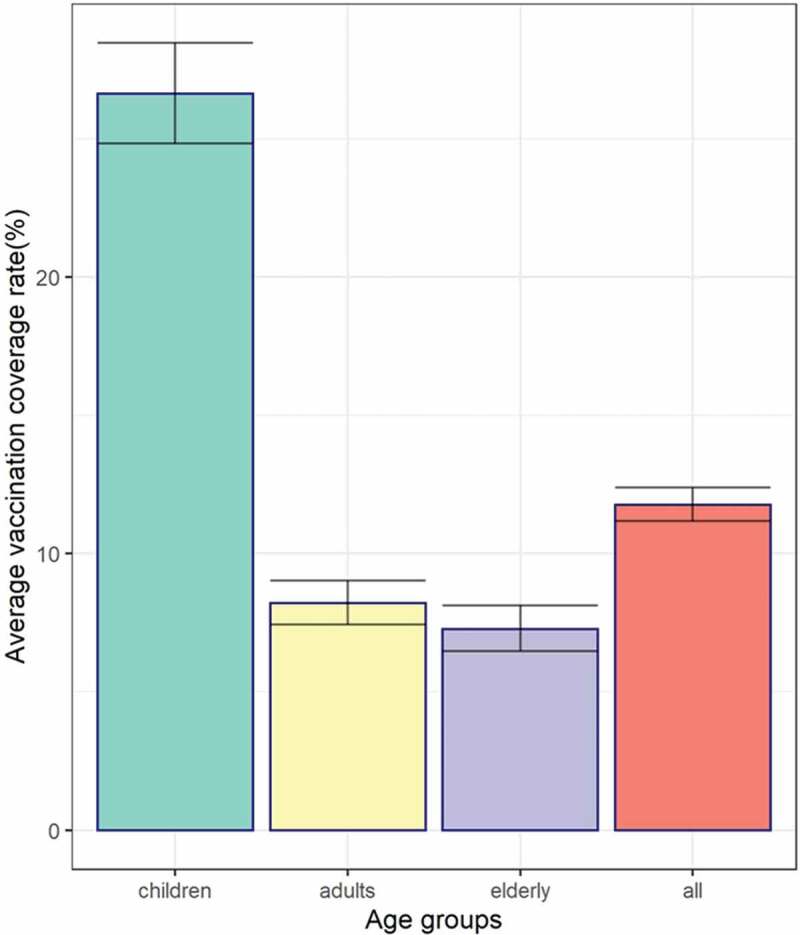
Coverage rates and 95% CI of seasonal influenza vaccination among different populations in Pudong New Area, Shanghai, in the 2018–2019 influenza season

A total of 1301 respondents received an influenza vaccination during the 2018–2019 season including 623 children, 396 adults and 282 elderly people, and the total vaccine coverage rate was 11.8% [95% confidence interval (CI): 11.2–12.4]. Vaccine coverage was highest among children (26.6%, 95%CI: 24.8–28.5), followed by adults (8.2%, 95%CI: 7.4–9.0) and lowest among elderly people (7.3%, 95%CI: 6.5–8.1) (*p* < .001). Those with chronic underlying conditions all had higher vaccine coverage compared with those without for different groups (*p* < .01); however, different results were found for all participants (*p* < .001) (Figure 2).

There was no statistically significant difference in VCR among males and females for children and elderly people. For adults, women had significantly higher VCRs, with an odds ratio (OR) of 1.49 (95%CI: 1.18–1.89, *p* < .01). Among children, nursery age children and school students had higher vaccine coverage rates than children not in school (OR = 2.66 and 1.55, respectively). However, no significant differences were found among different age groups for adults and older people. Children who were born premature had higher VCRs than other children, with an odds ratio (OR) of 2.04 (95%CI: 1.37–3.04, *p* < .001) (Table 2).

**Table 2.** Coverage rates of seasonal influenza vaccination among different populations in Pudong New Area, Shanghai, in the 2018–19 influenza season (n = 11053)

			OR		
Characteristics	No. of Vaccinated Individuals	VCR (95% CI)	Children	Adults	The elderly
Total	1301	11.8(11.2–12.4)			
Gender					
Male	603	11.5(10.6–12.4)	Ref	Ref	Ref
Female	698	12.0(11.2–12.9)	0.92(0.76–1.10)	1.49(1.18–1.89)**	1.22(0.95–1.57)
Residence address					
Shanghai	1080	11.5(10.9–12.2)	Ref	Ref	Ref
Other province in China	221	13.3(11.7–15.0)	1.11(0.86–1.38)	1.01(0.74–1.36)	0.51(0.25–1.01)
Age group^a^					
Group1	623	26.6(24.8–28.5)	Ref	0.76(0.52–1.11)	0.99(0.69–1.42)
Group2	396	8.2(7.4–9.0)	2.60(1.92–3.52)**	0.97(0.72–1.32)	1.05(0.75–1.47)
Group3	282	7.3(6.5–8.1)	1.55(1.17–2.05)**	Ref	Ref
Living status					
Live alone	24	6.6(4.2–9.6)	–	Ref	Ref
Live with family or friends	1277	11.9(11.3–12.6)	–	1.09(0.46–2.56)	0.95(0.57–1.59)
Education attainment^b^					
Primary school	95	5.4(4.4–6.5)	–	0.38(0.16–0.91)*	Ref
Secondary school^c^	159	7.4(6.3–8.6)	–	0.46(0.32–0.68)**	1.77(1.29–2.43)**
High school	80	5.8(4.6–7.2)	–	0.47(0.32–0.69)**	1.28(0.86–1.90)
Bachelor’s degree or above	281	10.4(9.3–11.6)	–	Ref	2.65(1.68–4.19)**
Self-evaluation of health situation					
Very healthy	243	13.5(12.0–15.2)	Ref	Ref	Ref
Healthy	676	12.2(11.4–13.1)	1.17(0.94–1.45)	1.13(0.81–1.57)	0.84(0.45–1.58)
Normal	342	10.1(9.1–11.2)	1.46(1.05–2.02)*	1.29(0.90–1.87)	0.98(0.52–1.83)
Unhealthy	40	11.4(8.3–15.2)	1.03(0.11–10.13)	2.22(0.79–6.19)	1.43(0.69–2.95)
Monthly household income, (Chinese yuan)^d^					
<2000	13	4.2(2.2–7.1)	–	0.32(0.04–2.39)	0.44(0.20–0.97)*
2000–4999	168	8.4(7.2–9.7)	0.88(0.57–1.33)	0.63(0.41–0.98)*	0.64(0.36–1.12)
5000–7999	163	13.5(11.6–15.6)	0.89(0.62–1.28)	0.85(0.58–1.23)	0.78(0.41–1.49)
≥8000	212	17.8(15.7–20.1)	Ref	Ref	Ref
Specific underlying diseases					
Premature	46	42.2(32.8–52.0)	2.04(1.37–3.04)**	–	–
Chronic respiratory diseases	96	16.3(13.4–19.5)	1.34(0.91–1.99)	1.18(0.61–2.25)	1.37(0.92–2.04)
Diabetes	55	6.7(5.1–8.7)	–	0.43(0.16–1.21)	0.90(0.65–1.25)
Cardiovascular disease	239	8.5(7.5–9.6)	5.18(0.91–29.41)*	1.43(0.98–2.07)	1.39(1.06–1.83)*

^a^Age group among total respondents referred to children(group1), adults (group2) and elderly(group3); Age group in children referred to scattered children (group1), nursery children (group2) and school students (group3) respectively; age group in adults referred to 15–29 years (group1), 30–44 years (group2) and 45–59 years (group3); age group in the elderly referred to 60–69 years (group1), 70–79 years (group2) and ≥80 years(group3). in children referred to scattered children, nursery children and school students respectively; age group in adults referred to 15–29 years, 30–44 years and 45–59 year s; age group in the elderly referred to 60–69 years, 70–79 years and ≥80 years.

^b^Children and students were not included.

^c^In China, secondary school education refers to the three-year period between primary school and high school. The ages of secondary school students are approximately 12 to 15 years old.

^d^1 US dollar6.43 Chinese yuan.

* *p* < 0.05; ** *p* < 0.01

Among adults, vaccine coverage was highest among those with bachelor’s degrees or above (10.1%, 95%CI: 9.0–11.4), followed by those with secondary school diploma (5.2%, 95%CI: 3.8–7.0) (*p* < .001). For the elderly, the highest and lowest vaccination rates were obtained among the respondents with bachelor’s degree or above (13.1%, 95% CI: 9.2–18.0) and those with a primary education only (5.4%, 95% CI: 4.4–6.7) (*p* < .001), respectively (Table 2).

There were no significant differences in the VCRs among individuals with chronic respiratory diseases and those with diabetes for all three groups. The VCRs among children and older people suffering from cardiovascular disease were significantly higher (*p* < .05) than the rates in the other groups (OR = 5.18, 1.39, respectively) (Table 2).

### Reasons for receiving or not receiving the influenza vaccine

**Figure 3.**
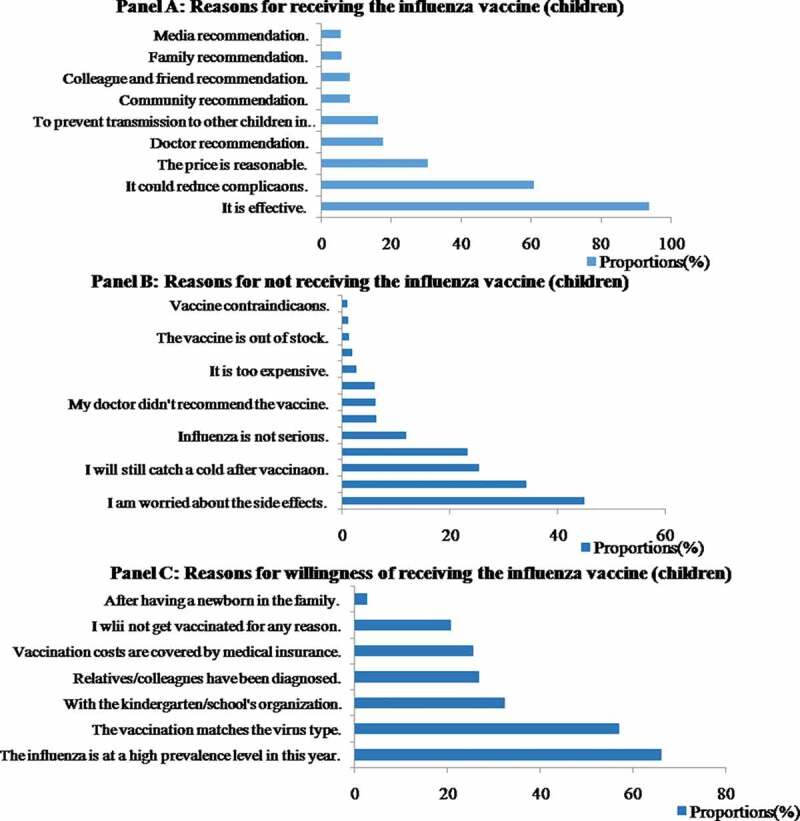
Coverage rates and 95% CI of seasonal influenza vaccination among different populations in Pudong New Area, Shanghai, in the 2018–2019 influenza season

**Figure 4.**
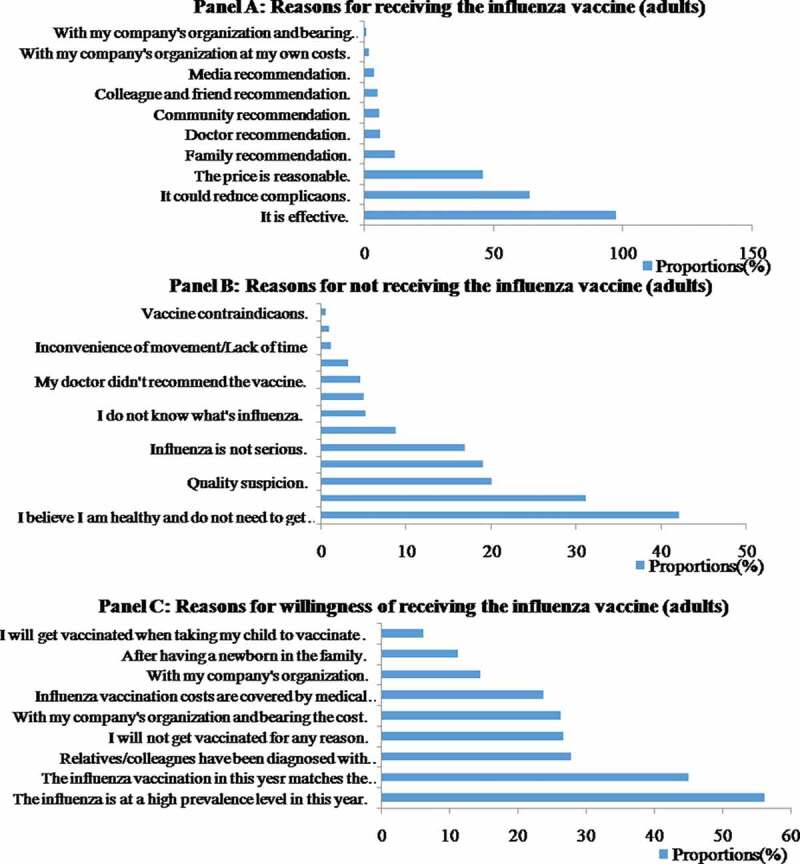
Reasons for receiving or not receiving the influenza vaccine among adults in Pudong New Area during the 2018–2019 influenza season

Among the 623 children, 396 adults and 282 elderly people who received an influenza vaccination during the 2018–2019 influenza season, most (93.9%, 97.5% and 92.9%, respectively) made this decision based on their belief that the influenza vaccine was effective (Figure 3A, 4A, 5A). The subsequent reason for the three groups was that they all believed the vaccination could reduce complications due to influenza (61.0%, 63.9%, 62.1%, respectively). A total of 30.7% of the vaccinated children and 46.0% of vaccinated adults received the vaccine because of the reasonable price, whereas 43.3% of the vaccinated elderly individuals stated that the reason for receiving the vaccination was that it was recommended by their family.

**Figure 5.**
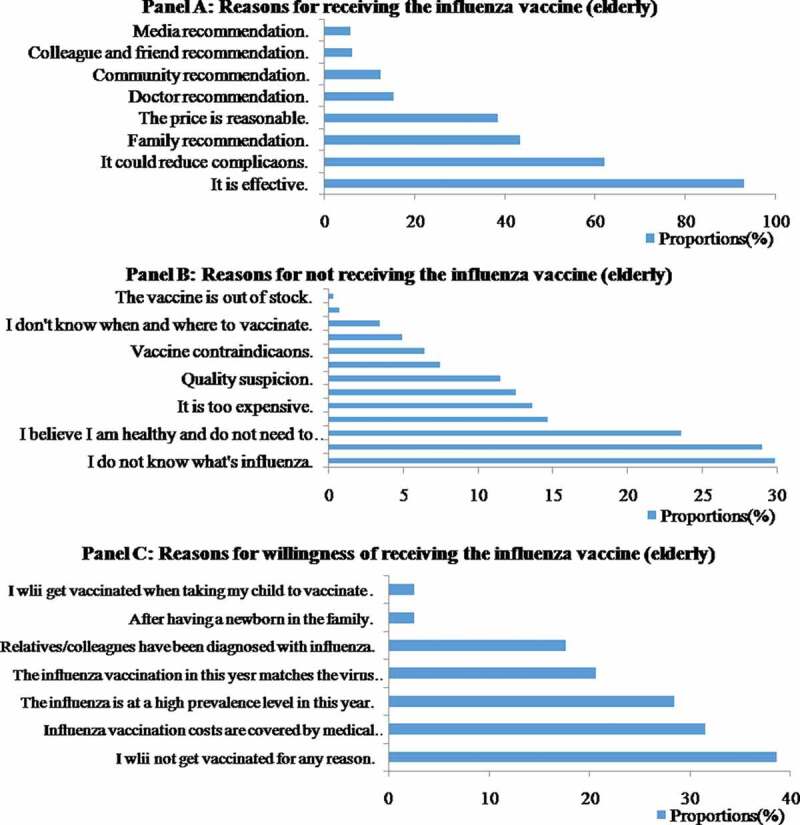
Reasons for receiving or not receiving the influenza vaccine among the elderly in Pudong New Area during the 2018–19 influenza season

For children, the main explanations for not being vaccinated were that their parents were worried about the side effects (45.0%), followed by suspicion about the vaccine quality (34.1%), and the belief that it was still possible to catch influenza after vaccination (25.4%). However, among the adults, most of the unvaccinated respondents claimed that they believed they were healthy and did not need to get vaccinated (42.2%), followed by worry regarding the side effects (31.2%), and suspicion about vaccine quality (20.3%). The main reason elderly people reported for not receiving influenza vaccine was lack of influenza or the vaccine awareness (29.8% and 28.9%). Other important reasons for not receiving vaccination among the elderly included a belief of being healthy enough and not needing to be vaccinated (23.6%) and believing that it was still possible to catch influenza after vaccination (14.7%) (Figure 3B, 4B, 5B).

### Willingness to vaccinate

We also investigated unvaccinated people who were willing to get vaccinated. The choices were also different among the three groups. Among children, nearly two-thirds (66.0%) would choose to get vaccinated if influenza was highly prevalent that year, followed by if they thought it was effective (57.0%), if the kindergarten/school was organized together (32.3%) and if relatives or people they knew were diagnosed with influenza. Among adults, more than half (56.3%) stated that their willingness to vaccinate would increase if they knew influenza was highly prevalent that year, followed by if they were convinced of the effectiveness of the vaccine (45.1%) and if relatives/colleagues had been diagnosed with influenza (27.8%). However, 26.7% of the adults claimed that they were unlikely to get vaccinated for any reason. Overall, willingness to vaccinate against influenza was low among the elderly. Nearly 40% of unvaccinated elderly participants (38.7%) stated they were unlikely to get vaccinated. A total of 31.5% of the older people were willing to get vaccinated if the costs were covered by medical insurance. Other factors that increased their willingness to vaccinate included whether influenza was highly prevalent that year (28.5%) and if they were convinced of the effectiveness of the vaccine (20.6%) (Figure 3C, 4C, 5C).

## Discussion

The area and population of Pudong New Area are nearly one-fifth of Shanghai and this survey was conducted in all 48 community health centers of the Pudong New Area, including centers in cities, urban-rural fringe areas and rural areas, which could be considered a random sample of Shanghai. The reported influenza vaccination coverage among surveyed participants for 2018/2019 season was 11.8% (95% CI: 11.2–12.4). This vaccination rate was similar to those of previous studies conducted in some cities of China.^7^ In our study, the elderly had the lowest vaccination rate in Shanghai, whereas the vaccination rate of adults (aged 18–59 years) was the lowest among previous studies conducted in some other cities of China.^7^ Perceptions among different populations may explain the age disparity and the older participants in our survey were even more lack of influenza vaccine awareness. Among the three different age groups, those with chronic underlying conditions all had higher vaccine coverage than those without; however, the opposite result was found for the total participants. The possible reason may be that there were more participants with chronic underlying conditions among the elderly, and the vaccination rate for the elderly was low, which lowered the overall level.

The reported VCRs among the three different age groups were lower than in other districts and countries, such as Hong Kong,^8^ the USA^9^ and some EU/EEA member states,^10^ especially the VCR among elderly people. The VCR rate of the elderly was not only markedly lower than the rates reported in European countries,^10^ the United States,^9^ Japan,^11^ Korea^12^ and Singapore,^13^ but it was also much lower than reported by studies in Beijing,^14^ which is similar in size to Shanghai.

The influenza VCRs among subgroups of children were similar, with the exception of the rates among nursery children and school students as well as the rates among premature babies, which were significantly higher than the VCRs obtained for the other groups. For adults, women had significantly higher influenza VCRs than men; a possible reason was that women may pay more attention to health. Similarly to other studies,^7^ this survey showed that education level may influence influenza vaccination rates. Higher education levels may correlate with learning more about influenza and vaccination.

Previous studies have shown that low vaccination rates may be associated with the influenza vaccination reimbursement strategy.^7,15^ The Chinese Center for Disease Prevention and Control recommends annual seasonal influenza vaccination for children aged six to 59 months, the elderly aged 60 years and older, persons with specific chronic medical conditions, health-care workers, family members and caregivers of babies under 6 months of age, and pregnant women, which is a bit different from the WHO recommendation^16,17^ However, the influenza vaccine is not currently included in the national Expanded Program on Immunization (EPI) in China. Since 2007, the governments of some cities in China such as Beijing,^14^ Karamay,^18^ and Xinxiang^19^ have published policies providing free influenza vaccinations to local elderly people. In addition, some areas of Guizhou and Zhejiang Province have implemented subsidy policies such as including the influenza vaccine in medical insurance for target groups.^20,21^ However, no such policies have been implemented in Shanghai. One study showed that the policy in Beijing greatly increased the vaccine uptake rate in the population qualifying for free vaccination.^22^ Similarly, 31.5% of the unvaccinated older people in this study showed their willingness to get vaccinated if the costs were covered by medical insurance.

At the same time, complicated vaccination policies have not only increased national vaccination rates but also brought great difficulties in management and control. Our results showed that the VCRs were greatly affected by suspicion of the quality of the vaccine. Regular and strict management could prevent vaccine-related events, such as the Changchun Longevity Vaccine Event.^23^ – DTP vaccines produced by Changchun Longevity (no. 201605014–01) did not meet quality standards, and the event involved 215,184 vaccinated children. They fabricated production records and product inspection records of rabies vaccines. Considering the economic burden of the influenza vaccine, more regular reimbursement policies need to be conducted in the future.

Recommendation from health-care workers (HCWs) may also effectively increase influenza vaccination rates.^15,24^ Not only was the vaccination coverage among HCWs in mainland China low,^25^ but the rate among HCWs who have actively recommended patients for influenza vaccination was also low.^24^ Similarly, 13.6% of the 1301 vaccinated respondents in this study stated that they received the vaccine due to a doctor’s recommendation. That the rate among populations was low may be consistent with the low vaccination rates among HCWs. Raising the influenza vaccine rate among HCWs may increase the vaccination rate among other populations.

We also found that another main explanation for not being vaccinated among children and adults was worrying about the side effects (34.5%). The main reason elderly people reported for not receiving influenza vaccine was a lack of awareness of inﬂuenza or the vaccine (29.8% and 28.9%). Therefore, being vaccinated might be influenced not only by local policy but could also be strongly affected by knowledge and attitudes toward the vaccine. This finding suggested that publicity strategies should be adopted according to the characteristics of the target population to achieve the best effect. Interventions should concentrate on strategies to inform people that vaccination is the most powerful protective measure against influenza and to treat the side effects rationally. One study revealed that mass information and communication was a further element to achieve high VCRs.^15^ Yoo et al. identified a positive relationship between high media coverage of annual awareness campaigns and vaccination rates.^15,26^ It seemed to be crucial to reinforce the disease and vaccine awareness of the public in order to increase vaccination rates. This can be done by, e.g., the media, websites or information provided in waiting rooms.

The WHO’s vaccine hesitancy influencing factors matrix are grouped in three categories: contextual, individual and group and vaccine/vaccination-specific influences.^27^ According to our results, vaccine/vaccination-specific influences were the most important determinants of vaccine hesitancy for children, including the risk of side effects and suspicion about quality. For adults and the elderly, individuals and groups had greater influences on vaccine hesitancy than did attitudes about health and prevention and knowledge of influenza and the vaccine, respectively.

The limitations of this study are similar to those associated with retrospective survey and cross-sectional study designs, including recall bias and selection bias. In addition, the limitation from convenience sampling may lead to bias in the generalization of the findings. Our survey was conducted among only older residents seeking medical services at Community Health Service Centers (CHSCs). Obviously, older adults who pay more attention to their health and who tend to seek primary health-care services have a greater opportunity for receiving information about the influenza vaccine and being vaccinated. Such people may have been over-sampled in our study. In contrast, those who seldom or never seek health services at CHSCs might not have been recruited for this survey. This selection bias might be the reason for the higher VCR from our study than those from previous studies in China.

## Conclusions

In this study, for the first time, we quantitatively revealed the influenza VCR in a large sample of different populations in Shanghai, China. Our study found that the overall influenza VCR is low in Shanghai, especially among older people. Low education level and poor awareness of this vaccine were the leading barriers to accepting the influenza vaccine; great efforts should be made according to the characteristics of the target population to enhance the perception of the influenza virus and influenza vaccine.
